# Medical Inspection of Schools

**Published:** 1905-02-04

**Authors:** 


### Medical Inspection of Schools.

We understand that some displeasure has been excited, in the minds of an eminently deserving body of public servants, by a leading article, which appeared in the *Times* of January 10th, and was based upon a recent address by Sir Lauder Brunton. The article referred to the medical inspection of schools for the purpose of discovering the earliest indications of infectious disease, and expressed the view that, in localities where the medical officer of health was debarred from private practice, although he should be the school authority in all sanitary matters, and should be supreme over drainage, ventilation, lighting, cubic space, and so forth, yet that it might be better for the authorities to employ a practising medical man as the visiting medical officer of the school in relation to any questions in which the health of individual children might be concerned. The suggestion was that the public health officer, debarred from practice, and thus cut off from personal contact with sick people, would be not unlikely to lose something of his original quickness of clinical perception, and of his original aptitude for dealing with individual patients. We are told that this assumption is somewhat warmly resented by some of the medical officers of health concerned, who are inclined to assert their entire fitness for dealing with clinical, as well as with administrative, questions, and to say that they are as well qualified as (if not better than) the local practitioner to decide whether a particular child is suffering from a condition which may be prejudicial to the rest. We think the gentlemen who thus argue, and to the excellence of whose special work, by the way, the *Times* paid a well-deserved tribute, are somewhat unduly sensitive. We think the fear that the man restricted from ordinary medical practice would in time lose somewhat of his aptitudes for diagnosis is not a visionary one, and he is still more likely—nay, still more certain—to lose something of his professional knack of dealing with sick children and of winning their confidence. In rural schools especially the medical officer of health would be a stranger of imposing aspect, brought from a distance to undertake an investigation, while the practitioner would be a neighbour whom the children were accustomed to see every day, and from whose inquiries they would not be so likely to shrink. Moreover, in many, if not in most, cases he would know the aspects of the individual children in health, an advantage which should certainly not be overlooked. His perceptions would be sharpened by his direct personal interest in avoiding an epidemic which would add to his cares without increasing his remuneration. On the whole, we think not only that the view taken by the *Times* is correct, but that its correctness is almost self-evident. If, in the case of any particular child, the practising and the non-practising doctor differed in their diagnosis there would be high probability, other things being equal, that the former would be right; for it follows from the ordinary conditions of human activity that what a man is always doing he will be likely to do well, and also, in regard of what he ceases to do, that he will not be unlikely to become rusty. Moreover, the local practitioner, if placed definitely in charge of the health of the school, would have a responsibility towards his own neighbours and patients which he would have every motive to justify. It is no disparagement of even the most eminent D.P.H. to say that there are departments of medicine in which practice tends to make perfect, and in which even conspicuous ability requires to be reinforced by the daily teachings of experience.

